# Survivin Selectively Modulates Genes Deregulated in Human Leukemia Stem Cells

**DOI:** 10.1155/2011/946936

**Published:** 2010-12-23

**Authors:** Seiji Fukuda, Mariko Abe, Chie Onishi, Takeshi Taketani, Jamiyan Purevsuren, Seiji Yamaguchi, Edward M. Conway, Louis M. Pelus

**Affiliations:** ^1^Department of Pediatrics, Shimane University School of Medicine, 89-1 Enya-Cho, Izumo, Shimane 693-8501, Japan; ^2^Department of Microbiology and Immunology, Indiana University School of Medicine, Indianapolis, IN 46202-5254, USA; ^3^Department of Hematology, Shimane University School of Medicine, Shimane 693-8501, Japan; ^4^Division of Blood Transfusion, Shimane University Hospital, Shimane 693-8501, Japan; ^5^Centre for Blood Research, University of British Columbia, Vancouver, BC, Canada V6T 1Z3

## Abstract

ITD-Flt3 mutations are detected in leukemia stem cells (LSCs) in acute myeloid leukemia (AML) patients. While antagonizing Survivin normalizes ITD-Flt3-induced acute leukemia, it also impairs hematopoietic stem cell (HSC) function, indicating that identification of differences in signaling pathways downstream of Survivin between LSC and HSC are crucial to develop selective Survivin-based therapeutic strategies for AML. Using a Survivin-deletion model, we identified 1,096 genes regulated by Survivin in ITD-Flt3-transformed c-kit^+^, Sca-1^+^, and lineage^neg^ (KSL) cells, of which 137 are deregulated in human LSC. Of the 137, 124 genes were regulated by Survivin exclusively in ITD-Flt3^+^ KSL cells but not in normal CD34^neg^ KSL cells. Survivin-regulated genes in LSC connect through a network associated with the epidermal growth factor receptor signaling pathway and falls into various functional categories independent of effects on apoptosis. Pathways downstream of Survivin in LSC that are distinct from HSC can be potentially targeted for selective anti-LSC therapy.

## 1. Introduction

Survivin has been implicated in regulation of apoptosis, cell division, and cell cycle both in cancer cells and normal tissues, through caspase-dependent and -independent mechanisms [1–3]. We previously showed that Survivin is expressed and growth factor regulated in human CD34^+^ cells [4–6]. Antagonizing Survivin impairs production of mouse bone marrow hematopoietic progenitor cells *in vitro * [4, 7] and conditional Survivin gene deletion *in vivo* in mice leads to bone marrow ablation as a result of loss of hematopoietic stem and progenitor cells (HSPC) [8]. In contrast to tight regulation of Survivin by hematopoietic growth factors in normal CD34^+^ cells, deregulated expression of Survivin is frequently observed in hematological diseases, particularly those associated with hematopoietic stem cell (HSC) expansion. For instance, Survivin is aberrantly overexpressed in acute myeloid leukemia [9, 10] but downregulated in marrow cells of patients with aplastic anemia where HSPC are significantly reduced [11]. These findings suggest that Survivin regulates HSPC fate under normal and pathological conditions. 

We previously reported that ITD-Flt3 mutations found in ~25–30% of patients with acute myeloid leukemia (AML) and strongly associated with poor prognosis [12–14], increase expression of Survivin. Survivin mediates aberrant hematopoietic cell proliferation induced by ITD-Flt3 and regulates development of ITD-Flt3^+^ acute leukemia, suggesting that antagonizing Survivin may provide therapeutic benefit for patients with AML expressing ITD-Flt3 [14]. Survivin is the fourth most highly expressed transcript in cancer [15] and is commonly associated with a higher proliferative index, reduced apoptosis, resistance to chemotherapy and increased rate of tumor recurrence in cancer cells, making anti-Survivin therapy an attractive strategy in cancer [3]. Several anti-Survivin preclinical trials in solid tumor models show that disrupting Survivin can reduce tumor growth [1–3]. However, studies from our group and others indicate that Survivin regulates normal HSPC [4, 7, 8], suggesting that targeting Survivin will likely result in hematopoietic toxicity. Therefore, identification of differences in signaling cascades downstream of Survivin between normal HSPC and cancer stem cells (CSC) or leukemia stem cells (LSC) are required to pinpoint targets that can effectively eradicate CSC/LSC with little toxicity on HSC. Previous reports show that ITD-Flt3 mutations are present in human LSC [13] and genes expressed in AML stem cells are deregulated [16]. The primary purpose of this study was to identify downstream Survivin signaling pathways in LSC that are distinct from normal HSC. Using ITD-Flt3 transformed c-kit^+^, Sca-1^+^, and lineage^neg^ (KSL) cells from conditional Survivin knockout mice as surrogates for AML stem cells and littermate controls, we identified a panel of genes that are specifically regulated by Survivin in ITD-Flt3 transformed KSL cells and known to be deregulated in human LSC. The data identify selective signaling pathways downstream of Survivin in LSC that are distinct from normal HSC that can be potentially targeted for selective anti-LSC therapy.

## 2. Materials and Methods

### 2.1. Antibodies and Cytokines

Anti-Fc*γ*-III/II receptor antibody, APC conjugated antimouse c-kit (clone 2B8), biotin-conjugated anti-Sca-1 (E13-161.7), R-Phycoerythrin (PE) conjugated anti-mouse CD3 (clone 143-2C11), GR-1 (clone RB6-8C5), B220 (clone RA3-6B2), Mac1 (clone M1/70), Ter119 (clone Ter119), rat IgG2a, rat IgG2b, hamster IgG, and Streptavidin-PE-Cy7 were purchased from BD Biosciences (San Diego, CA). Recombinant human Flt3 ligand (FL) and Thrombopoietin (Tpo) were provided by Amgen, Thousand Oaks, CA. Recombinant murine stem cell factor (rmSCF) was purchased from R&D Systems (Minneapolis, MN). Tamoxifen and 4-hydroxy (4OH) tamoxifen were from Sigma-Aldrich (St. Louis, MO).

### 2.2. Cell Culture, Plasmid Transfection, Retrovirus Transduction, and In Vitro Survivin Gene Deletion

Mice with the Survivin gene flanked by loxP sites and a Tamoxifen-inducible form of Cre (Cre-ER) were reported previously [14]. All mice were housed in microisolators with continuous access to rodent chow and acidified water. The Indiana University School of Medicine IACUC approved all experimental procedures. Retrovirus transduction of ITD-Flt3 into mouse bone marrow cells was carried out as described [14]. Briefly, bone marrow cells from littermate control Survivin^flox/flox^ or CreER-Survivin^flox/flox^ mice were transduced with ITD-Flt3 (N51) in MSCV-IRES-EGFP vector [17]. Seventy-two hours after sequential transduction, the medium was replaced with IMDM containing 10% FBS and the cells were incubated for 14 days without growth factors in the presence of 1uM of 4OH-Tamoxifen to induce Survivin gene deletion. All cells were cultured in triplicates in two independent experiments. Viable cells were enumerated using trypan blue exclusion and GFP^+^, c-kit^+^, Sca-1^+^, and lineage negative (KSL) cells were sorted on day 14 by FACSAria (BD Biosciences). Sorted cells were immediately lysed and subjected to differential mRNA microarray analysis.

### 2.3. In Vivo Survivin Deletion

Survivin^flox/flox^ and CreER-Survivin^flox/flox^ mice were treated with 5 mg Tamoxifen (5 mg/mouse i.p.) for 3 consecutive days and allowed to rest for 3 days, and Tamoxifen was administered for 3 additional days. Fourteen days after the final Tamoxifen injection, marrow cells were harvested, stained with anti-CD34, c-kit, Sca-1, and lineage markers and CD34^neg^ KSL cells isolated by FACSAria for mRNA microarray analysis.

### 2.4. mRNA Microarray

Sorted cells were immediately lysed and subjected to differential mRNA microarray analysis. Microarray analysis was performed on a fee basis by Miltenyi Biotec (Auburn, CA). Briefly, 250 nanograms of each cDNA were used as template for Cy3 and Cy5 labeling which was performed according to Miltenyi Biotec's proprietary protocol. Equal amounts of labeled cDNAs from Tamoxifen-treated Survivin^flox/flox^ and CreER-Survivin^flox/flox^ mice or those cells expressing ITD-Flt3 were hybridized overnight (17 hours, 65°C) to Agilent Whole Mouse Genome Oligo Microarrays (44 K) according to the manufacturers' protocol. Fluorescence signals of the hybridized microarrays were detected using Agilent's DNA microarray scanner (Agilent, Palo Alto, USA). The Agilent Feature Extraction Software (FES) was used to read out and process the microarray image files. The software determines feature intensities and ratios (including background subtraction and normalization), rejects outliers, and calculates statistical confidences (*P*-values). For determination of differential gene expression, FES derived output data files were further analyzed using the Rosetta Resolverâ gene expression data analysis system (Rosetta Biosoftware, Seattle, USA). Significantly regulated genes were annotated and assigned to functional categories using the DAVID 2008 (the Database for Annotation, Visualization and Integrated Discovery; http://david.abcc.ncifcrf.gov/home.jsp) program [18]. Functional networks of the genes regulated by Survivin was visualized using Cytoscape software: http://www.cytoscape.org/ [19].

## 3. Results

### 3.1. Reduction in Proliferation ITD-Flt3 Transformed KSL Cells by Survivin Deletion Is Associated with Alteration of Expression of 1,096 Genes Classified into Various Biological Functions

**Figure 1:**
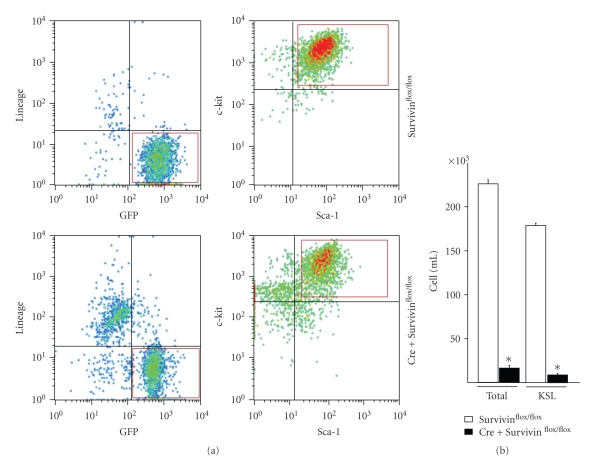
The effect of Survivin deletion on the number of KSL cells transformed by ITD-Flt3 (a) The KSL cells transformed by ITD-Flt3 were FACS sorted using GFP as a marker. Marrow cells derived from littermate control Survivin^flox/flox^ or CreER-Survivin^flox/flox^ mice were transduced with ITD-Flt3 (N51) in MSCV-IRES-EGFP vector. Seventy-two hours after transduction, the medium was replaced with IMDM containing 10%FBS and the cells were incubated for 14 days in the presence of 1uM of 4OH-Tamoxifen without hematopoietic growth factors. GFP^+^, c-kit^+^, Sca-1^+^, and lineage-negative (KSL) cells shown in red box were sorted on day 14 by FACSAria (BD Biosciences). (b) The number of whole cells and KSL cells derived from Survivin^flox/flox^ or CreER-Survivin^flox/flox^ marrow cells transduced with ITD-Flt3 cultured with 4OH-tamoxifen in the absence of any growth factors was quantitiated in triplicate wells of each sample averaged from 2 independent experiments. *P < .02 compared to control Survivin^flox/flox^.

While incubation of marrow cells transduced with wild-type Flt3 in IMDM containing 10% FBS without hematopoietic any growth factors failed to support KSL proliferation, overexpression of ITD-Flt3-EGFP in primary mouse bone marrow cells results in the ability of KSL cells to proliferate in the same culture condition without additional any hematopoietic growth factors as we reported, indicating that ITD-Flt3 is sufficient to transform primitive HSPC [14]. Incubation of ITD-Flt3 transduced marrow cells derived from CreER-Survivin^flox/flox^ mice with 1uM 4OH-Tamoxifen significantly reduced factor-independent KSL cells on day 14 compared to Survivin^flox/flox^ cells treated in exactly the same manner (Figure 1), confirming that Survivin is required for factor independent growth of ITD-Flt3 transformed KSL cells.

**Figure 2:**
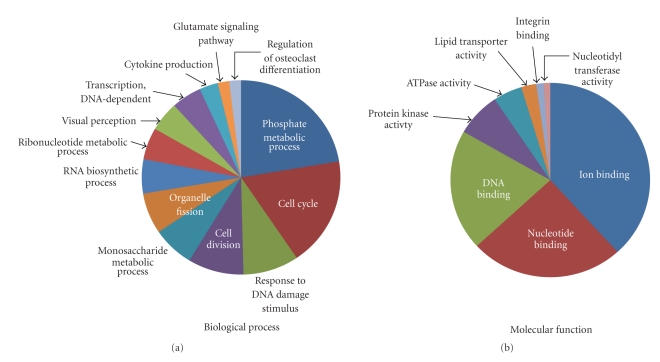
Classification of the Survivin regulated genes in ITD-Flt3 transformed KSL cells. The 1,096 genes identified by Survivin gene deletion were classified into biological process (a) and molecular function (b) defined by Gene Ontology (GO) Term using DAVID program (*P* < .02). Genes for which annotations could not be assigned were excluded. Representative functional categories are shown in the figures because of redundancies in classification categories. The complete Gene ID and annotations are listed in Supplementary Table S1 available at doi:10.1155/2011/946936.

To investigate the mechanism by which Survivin regulates aberrant proliferation of ITD-Flt3 transformed KSL cells, mRNA expression was compared between ITD-Flt3 transformed Survivin^flox/flox^ and CreER-Survivin^flox/flox^ KSL using mRNA microarrays. Although inhibition of Survivin mRNA expression in CreER-Survivin^flox/flox^ KSL cells by 4OH-Tamoxifen was only 50% compared to control, the mRNA microarray identified 1,096 transcripts differentially regulated by Survivin. These genes were classified based on biological process and molecular function defined by Gene Ontology Term (http://www.geneontology.org/) using DAVID 2008 program (Figure 2). Representative groups for biological process include phosphate metabolic process, cell cycle, cell division, response to DNA damage stimulus, RNA biosynthetic process and transcription (Figure 2(a), *P* < .02). Ion-binding, nucleotide-binding, DNA-binding, and protein kinase activity were the top 4 significantly enriched categories in molecular function defined by Gene Ontology database (Figure 2(b), *P* < .02). The list of genes classified by biological process and molecular function are listed in supplementary Table S1 available at doi:10.1155/2011/946936.

### 3.2. Survivin Modulates Expression of Genes Deregulated in Human AML LSC

**Figure 3:**
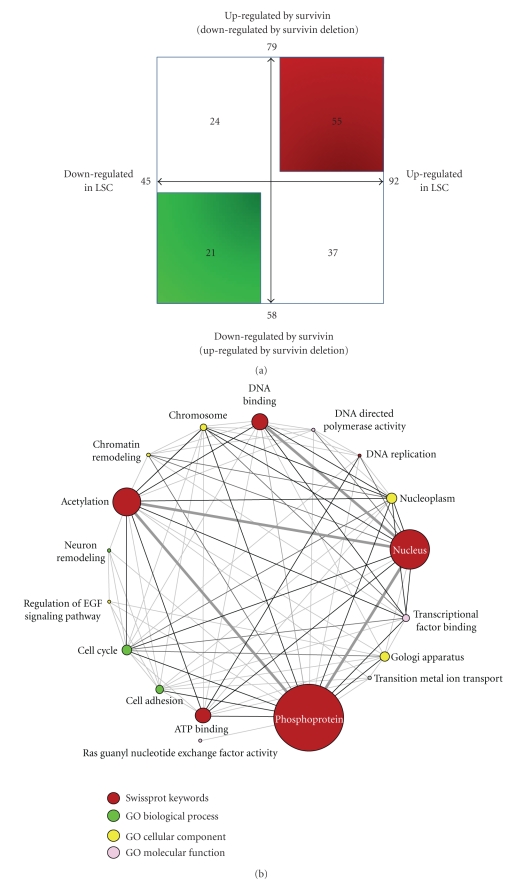

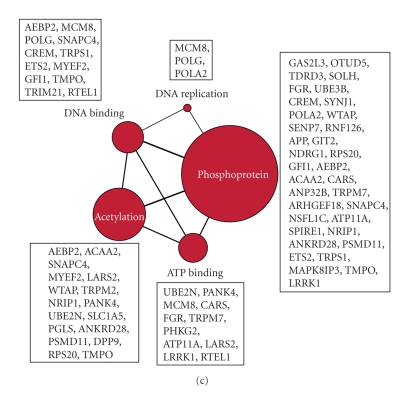
Classification of genes regulated by Survivin and LSC. (a) The 137 genes were classified based on the regulation by human LSC and Survivin in ITD-Flt3^+^KSL cells. Arrow bars in the quadrant indicate the direction of gene regulation by Survivin or LSC. The top-right corner shows 55 genes upregulated by both LSC and Survivin (downregulated by Survivin gene deletion), whereas bottom-left corner represents 21 genes downregulated by both LSC and Survivin (upregulated by Survivin gene deletion). The top left corner shows 24 genes downregulated by LSC but upregulated by Survivin. The bottom-right corner indicates 37 genes upregulated by LSC but downregulated by Survivin. (b) Functional group association networks for Survivin regulated genes in the list of deregulated genes in human LSC visualized using Cytoscape program. The 137 genes listed in Tables 1(a)–1(d) were functionally annotated using DAVID software (*P* < .05). The size of each circle represents the number of genes involved in each functional categories and the thickness of the line indicates the number of genes shared with any function. Functional classification was performed based on Gene Ontology Term database (biological process, molecular function and cellular component) and Swissprot Keywords. (c) The 76 genes upregulated or downregulated by Survivin and LSC (55 + 21 genes shown in Figure 3(a)) were functionally classified by DAVID software and visualized by Cytoscapse program as shown in Figure 3(b). The size of each circle represents the number of genes involved in each functional categories and the thickness of the line indicates the number of genes shared with any function. Functional classification was performed based on Swissprot Keywords. Genes annotated in each functional category are shown in the box.

The requirement of Survivin for the self-renewing capability of ITD-Flt3 transformed KSL cells or CFU [14] and the presence of ITD-Flt3 in LSC [13] strongly suggests that Survivin is important for LSC fate decisions resulting from deregulated gene expression. We compared 1,096 differentially expressed genes in Survivin deleted ITD-Flt3^+^KSL cells with the existing deregulated gene expression database for human AML stem cells [16]. Out of 1,096 genes regulated by Survivin in KSL cells transformed by ITD-Flt3, 137 genes are also listed in the deregulated molecules in human AML stem cells (Tables 1(a)–1(d)) [16]. The relationship between Survivin and LSC on the modulation of the gene expression is illustrated in Figure 3(a). Of the 137 genes identified, 79 genes were downregulated by Survivin deletion while 58 genes were upregulated, implying that these genes are conversely increased and decreased by the presence of Survivin in LSC (Figure 3(a)). In contrast, 92 genes were upregulated and 45 genes were downregulated by LSC. Among the 79 genes downregulated genes by Survivin deletion, 55 transcripts were upregulated (Figure 3(a) and Table 1(a)) whereas 24 genes were downregulated by LSC (Figure 3(a) and Table 1(c)). Similarly, out of 58 upregulated transcripts by Survivin deletion, 21 genes were downregulated (Figure 3(a) and Table 1(b)) and 37 genes were upregulated in LSC (Figure 3(a) and Table 1(d)) (Figure 3(a)). 

Next, we classified the 137 genes into functional annotation groups using the DAVID program (*P* <.05, Figure 3(b)). Significantly enriched functional groups were annotated as phosphoprotein, nucleus, acetylation, cell cycle, ATP binding, regulation of EGF signaling pathway, cell adhesion and others. The size of each circle in Figure 3(b) represents the number of genes involved in each functional category and the thickness of the line indicates the number of genes shared with any function, suggesting enrichment of the gene groups with functional redundancy. Among the molecules most frequently shared within the functional groups include BGLAP, Chrac1, Hmgb1 and Smarce1. Similarly, 76 genes upregulated or downregulated both by Survivin and LSC (55 + 21 genes shown in Figure 1(a)) were functionally classified (Figure 3(c)). Phosphoprotein, acetylation, DNA binding, ATP binding and DNA replication were significantly enriched.

The 1,096 differentially Survivin-regulated genes were also searched against the list of genes expressed in mouse HSC [20]. We identified 94 differentially expressed genes in the list of genes that include HSC. There were 45 genes whose expression was selectively enriched in HSC compared to other populations (Table 2). Crem, Emp1, Hmga2, Lrrn1, Maff, Myef2, Rps4x, and Sos1 listed in HSC database are also deregulated in LSC. We next compared the human LSC associated 137 genes regulated by Survivin in ITD-Flt3^+^KSL cells with differentially regulated genes in normal bone marrow CD34^neg^ KSL cells obtained from CreER-Survivin^flox/flox^ and control Survivin^flox/flox^ mice following Survivin deletion. Tamoxifen reduced Survivin expression by 10-fold in CD34^neg^ KSL cells from CreER-Survivin^flox/flox^ mice compared to control Survivin^flox/flox^ in two independent experiments. Out of 137 genes, Arg2, Med25, Pmaip1, Pola2, Ube3b, Ephb2 and Rab18 were differentially regulated in both ITD-Flt3^+^KSL cells and normal CD34^neg^ KLS cells by Survivin deletion. In contrast, Cenpa, Cpd, Myef2, Nmt2, Taf1b and Tmpo were downregulated in ITD-Flt3^+^KSL cells while they were upregulated in normal CD34^neg^ KSL cells by Survivin deletion. These findings suggest that the 124 genes are regulated by Survivin exclusively in ITD-Flt3^+^KSL cells but not in CD34^neg^ KSL cells. The complete list of the genes regulated by Survivin in CD34^neg^ KSL cells will be reported elsewhere (manuscript in preparation).

### 3.3. Survivin Modulates Gene Expression in LSC That Connects Through a Functional Signaling Network Associated with Epidermal Growth Factor Receptor Signaling Pathway

**Figure 4:**
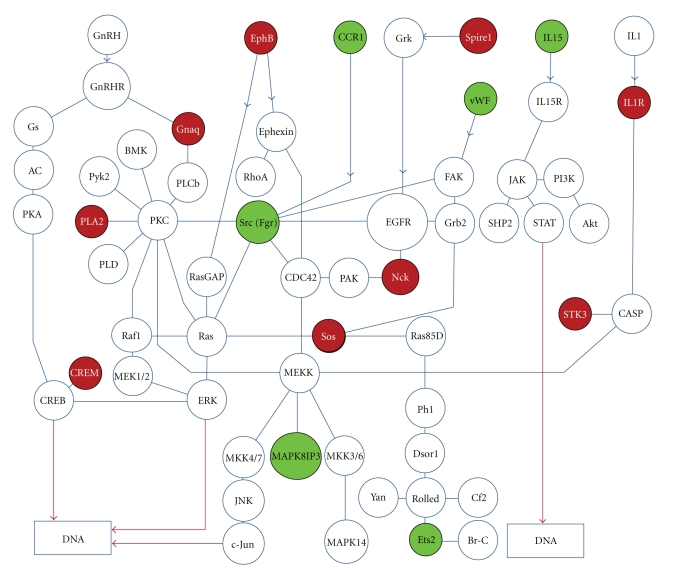
Genes regulated by Survivin in ITD-Flt3^+^KSL cells and deregulated in LSC are mapped on functional signaling network associated with epidermal growth factor receptor signaling pathway. Epidermal growth factor receptor signaling pathway (EGFR) is significantly enriched in the shared genes associated with LSC and Survivin signaling by pathway enrichment analysis in KEGG and Gene Ontology database. Our analysis shows that 13 molecules shared by LSC and Survivin signaling are mapped on a functional signaling network associated with EGFR. The network was created based on KEGG database using Cytoscape software. Green circle and red circle represents downregulation and upregulation by Survivin gene deletion, respectively.

Functional annotation analysis indicates that genes related with dorsoventral axis formation or epidermal growth factor receptor signaling pathway (EGFR) are significantly enriched in the shared genes associated with LSC and Survivin signaling in KEGG database (http://www.genome.jp/kegg/) (*P* < .03). Similarly, genes associated with regulation of EGFR signaling pathway are enriched in the Gene Ontology database (*P* < .02). Our analysis shows that 13 molecules shared by LSC and Survivin signaling are mapped on a functional signaling network that connects through the EGFR pathway (Figure 4).

## 4. Discussion

Survivin deletion in ITD-Flt3 transformed KSL cells results in a significant reduction in growth factor-independent proliferation coincident with growth inhibition. Our data shows that Survivin deletion modulates gene expression of 1,096 genes that are associated with various cellular and metabolic functions. Comparison of the 1,096 Survivin regulated genes in ITD-Flt3^+^KSL cells with 3,005 differentially regulated genes in the human AML stem cell database identified 137 shared genes. Functional classification of these 137 genes indicates they connect through a functional signaling network associated with EGFR signaling pathway and affect various biological and molecular processes. Comparison of the 137 Survivin-regulated genes in LSC with transcripts regulated by Survivin in normal CD34^neg^ KSL cells show that 124 genes are regulated by Survivin exclusively in ITD-Flt3^+^KSL cells and not in normal HSC. These data suggest that Survivin contributes to deregulation of gene expression in AML stem cells via selective signaling pathways distinct from normal HSC that can be potentially targeted for therapeutic benefit.

There is no direct evidence that Survivin directly regulates gene transcription; however, modulation of Survivin can clearly affect transcription in cancer cells [21–23] and transgenic expression of Survivin alters the expression of multiple genes in the bladder [24]. 

 The involvement of Survivin in transcriptional regulation is consistent with the fact that Bir1, a *Caenorhabditis elegans* homologue of Survivin, regulates transcription, most likely through Histone phosphorylation by Aurora kinase [25]. Survivin is essential for activation of Aurora kinase that phosphorylates Histone H3 [26, 27], an event required for transcriptional regulation [28–30] and cytokinesis [31], suggesting that Survivin may regulate transcription via Aurora kinase mediated Histone H3 phosphorylation. Over expression of Survivin also leads to phosphorylation of the Sp1 transcription factor [21]. Since microarray analysis was performed on lineage marker negative viable cells that express GFP, c-kit, and Sca-1 and survived in culture, it is unlikely that the effects of Survivin on gene expression is a consequence of KSL apoptosis, although we cannot rule out indirect effects resulting from cell cycle arrest as a consequence of Survivin deletion. 

Survivin is known to inhibit caspases 3, 7, and 10 that mediate apoptosis [1–3]. Our functional annotation analysis of the genes regulated by Survivin indicates that Survivin modulates a number of genes that affect multiple biological processes and molecular functions, although inhibition of Survivin mRNA expression in CreER-Survivin^flox/flox^ KSL cells by 4OH-Tamoxifen was only 50% compared to control. While incompleteness of the gene deletion may affect the downstream signals, significant reduction of total cell number was observed. Since Survivin deletion induces cell death, the deleted cells are likely no longer present in the culture at the time of cell harvest for analyses, resulting in an underestimate of the efficiency of Survivin gene deletion and likely represents Survivin expression in cells that have escaped the Tamoxifen-induced deletion. The genes affected by Survivin deletion were classified into cell cycle, adhesion, DNA replication, transcriptional factor binding, acetylation, phosphorylation, and polymerase. We did not observe changes in genes that mediate apoptosis. These results suggest that Survivin can regulate ITD-Flt3 transformed KSL cell fate independent of its activity as a caspase inhibitor. More importantly, comparison of the 1,096 Survivin regulated genes in ITD-Flt3^+^KLS cells with the human AML stem cell database demonstrates that 137 genes (12.4%) modulated by Survivin are also deregulated in LSCs. Functional annotation analysis indicates that genes related to dorsoventral axis formation or epidermal growth factor receptor signaling pathway (EGFR) are significantly enriched in the shared genes associated with LSC and Survivin signaling, even though the number of molecules detected that associates with EGFR signaling is small. EGFR signaling activates signaling cascades involved in cell proliferation, such as Src, Sos, and MAP-kinases, and is known to be dysregulated in solid tumors [32, 33]. Our data also indicates that Survivin modulates expression of several genes that connect through the EGFR signaling pathway (Figure 4), suggesting a potential role of EGFR signaling downstream of Survivin in AML stem cells, despite the fact that EGFR is not usually upregulated in AML cells [34]. However, recent studies indicate an antineoplastic effect of an EGFR inhibitor in AML via off-target effects [34, 35]. Thus, our data would support EGFR signaling as a candidate pathway for treatment of patients with AML. 

While Survivin is not listed in the LSC database [16], it is expressed at higher levels in AML cells compared to normal CD34^+^ cells [9, 10]. Our data clearly show that Survivin affects expression of 137 genes associated with LSC and reduces aberrant KSL proliferation induced by ITD-Flt3. This finding suggests that antagonizing Survivin in LSC may normalize expression of the deregulated genes, which in turn can inhibit their aberrant proliferation. Since Survivin is expressed and regulated in normal CD34^+^ cells [4, 6] and antagonizing Survivin reduces normal HSPC *in vivo* [8], it is likely that Survivin deletion will affect normal HSC in the patients. Although direct comparison of gene expression in *in vitro* ITD-Flt3^+^KSL cells with *in vivo* CD34^neg^ KSL cells may not necessarily provide definite differences in Survivin signaling between these populations *in vivo*, we found that 124 out of 137 Survivin associated genes shared by ITD-Flt3^+^KSL cells and human LSC were not regulated in normal CD34^neg^ KSL cells by Survivin. This disparity of downstream signaling of Survivin between normal and transformed HSC may represent as a means of identifying novel therapeutic targets against LSC that would thereby supplant direct Survivin disruption which would otherwise toxic to normal HSC.

## 5. Conclusion

We identified genes regulated by Survivin in ITD-Flt3 transformed KSL cells that are deregulated in human AML stem cells that are distinct from normal HSC. This study will facilitate the identification of specific therapeutic targets downstream of Survivin to eradicate LSC without affecting normal HSC.

## Supplementary Material

The list of genes regulated by Survivin and classified based on GO biological process or GO molecular function.

## Figures and Tables

**Table:** (a) Genes upregulated in LSC but downregulated in ITD-Flt3^+^KSL cells deleted with Survivin (55 genes).

Symbol	Name	Fold change
Acaa2	acetyl-Coenzyme A acyltransferase 2 (mitochondrial 3-oxoacyl-Coenzyme A thiolase) (Acaa2), [NM_177470]	−3.7
Ankrd12	Mus musculus 15 days embryo head cDNA, RIKEN full-length enriched library, clone:D930020E11 product: unknown EST, full insert sequence. [AK086311]	−5.5
Ankrd28	Mus musculus 2 days neonate thymus thymic cells cDNA, RIKEN full-length enriched library, clone:E430019N21 product: unknown EST, full insert sequence. [AK088541]	−2.9
Ap1g2	adaptor protein complex AP-1, gamma 2 subunit (Ap1g2), [NM_007455]	−5.2
Arhgef18	rho/rac guanine nucleotide exchange factor (GEF) 18 (Arhgef18), [NM_133962]	−3.6
Arrdc1	arrestin domain containing 1 (Arrdc1), [NM_178408]	−2.1
Atp11a	ATPase, class VI, type 11A, [AK006628]	−2.6
BGLAP	bone gamma carboxyglutamate protein 1 (Bglap1), [NM_007541]	−10.2
Cars	cysteinyl-tRNA synthetase (Cars), [NM_013742]	−2.3
Ccr1	chemokine (C-C motif) receptor 1 (Ccr1), [NM_009912]	−3.5
Cd96	CD96 antigen (Cd96), [NM_032465]	−2.1
Cfp	complement factor properdin (Cfp), [NM_008823]	−4.3
Cyyr1	cysteine and tyrosine-rich protein 1 (Cyyr1), [NM_144853]	−2.7
Dpagt1	dolichyl-phosphate (UDP-N-acetylglucosamine) acetylglucosaminephosphotransferase 1 (GlcNAc-1-P transferase) (Dpagt1), [NM_007875]	−2.8
Dpp9	dipeptidylpeptidase 9 (Dpp9), [NM_172624]	−2.4
Elovl1	elongation of very long chain fatty acids (FEN1/Elo2, SUR4/Elo3, yeast)-like 1 (Elovl1), [NM_019422]	−2.5
Ets2	E26 avian leukemia oncogene 2, 3′ domain (Ets2), [NM_011809]	−2.5
Fgr	Mus musculus Gardner-Rasheed feline sarcoma viral (Fgr) oncogene homolog (Fgr), mRNA [NM_010208]	−2.9
Gfi1	growth factor independent 1 (Gfi1), [NM_010278]	−2.7
Gga2	golgi associated, gamma adaptin ear containing, ARF binding protein 2 (Gga2), [NM_028758]	−2.5
Git2	G protein-coupled receptor kinase-interactor 2, [AK017943]	−3.1
Hdac10	histone deacetylase 10 (Hdac10), [NM_199198]	−4.1
Ipo13	importin 13 (Ipo13), [NM_146152]	−2.3
Isyna1	myo-inositol 1-phosphate synthase A1 (Isyna1), [NM_023627]	−2.5
Klhl17	kelch-like 17 (Drosophila) (Klhl17), [NM_198305]	−4.8
Lars2	leucyl-tRNA synthetase, mitochondrial (Lars2), nuclear gene encoding mitochondrial protein, [NM_153168]	−3.6
Lrrk1	leucine-rich repeat kinase 1 (Lrrk1), [NM_146191]	−2.7
Lztr1	leucine-zipper-like transcriptional regulator, 1 (Lztr1), [NM_025808]	−3.4
Mapk8ip3	mitogen-activated protein kinase 8 interacting protein 3 (Mapk8ip3), [NM_013931]	−2.9
Mcm8	minichromosome maintenance deficient 8 (S. cerevisiae) (Mcm8), [NM_025676]	−2.4
Med25	TCBAP0758 protein (Fragment) homolog [Homo sapiens], [AK165760]	−5.4
Mr1	laminin receptor 1 (ribosomal protein SA) (Lamr1), [NM_011029]	−2.6
Napa	N-ethylmaleimide sensitive fusion protein attachment protein alpha (Napa), [NM_025898]	−3.0
Ndrg1	N-myc downstream regulated gene 1 (Ndrg1), [NM_010884]	−3.3
Nsfl1c	NSFL1 (p97) cofactor (p47) (Nsfl1c), [NM_198326]	−2.2
Otud5	bZ30I22.1 (Novel protein similar to human FLJ12550) (Fragment) homolog [Brachydanio rerio], [AK169921]	−3.1
Pank4	pantothenate kinase 4 (Pank4), [NM_172990]	−3.0
Pgls	6-phosphogluconolactonase, [AK158308]	−5.6
Phkg2	phosphorylase kinase, gamma 2 (testis) (Phkg2), [NM_026888]	−3.3
Pmaip1	phorbol-12-myristate-13-acetate-induced protein 1 (Pmaip1), [NM_021451]	−6.6
Pola2	polymerase (DNA directed), alpha 2 (Pola2), [NM_008893]	−2.7
Polg	polymerase (DNA directed), gamma 2, accessory subunit (Polg2), [NM_015810]	−3.2
Rnf126	ring finger protein 126 (Rnf126), [NM_144528]	−2.1
Ropn1l	ropporin 1-like (Ropn1l), [NM_145852]	−6.8
Rtel1	regulator of telomere elongation helicase 1 (Rtel1), [NM_001001882]	−4.3
Slc1a5	solute carrier family 1 (neutral amino acid transporter), member 5 (Slc1a5), [NM_009201]	−2.3
Slc25a28	solute carrier family 25, member 28 (Slc25a28), [NM_145156]	−2.9
Snapc4	small nuclear RNA activating complex, polypeptide 4 (Snapc4), [NM_172339]	−2.7
Solh	small optic lobes homolog (Drosophila) (Solh), [NM_015830]	−3.9
Synj1	PREDICTED: synaptojanin 1 (Synj1), [XM_358889]	−2.8
Trim21	tripartite motif protein 21 (Trim21), [NM_009277]	−11.4
Trpm2	transient receptor potential cation channel, subfamily M, member 2 (Trpm2), [NM_138301]	−4.2
Trpm7	transient receptor potential cation channel, subfamily M, member 7 (Trpm7), [NM_021450]	−3.2
Ube3b	ubiquitin protein ligase E3B (Ube3b), [NM_054093]	−2.8
Upp1	uridine phosphorylase 1 (Upp1), [NM_009477]	−3.4

Fold change: ITD-Flt3^+^ KLS cells derived from CreER-Survivin^fx/fx^ compared to control Survivin^fx/fx^ in two experiments.

**Table:** (b) Genes downregulated in LSC but upregulated in ITD-Flt3^+^KSL cells deleted with Survivin (21 genes)

symbol	Name	Fold change
Aebp2	AE binding protein 2 (Aebp2), transcript variant 3, [NM_009637]	11. 4
Anp32b	unspliced dna for: PROLIFERATION RELATED ACIDIC LEUCINE RICH PROTEIN PAL31 (SIMILAR TO ACIDIC PROTEIN RICH IN LEUCINES) homolog [Mus musculus],…	2.9
App	amyloid beta protein precursor, [M18373]	25.7
Cd164	CD164 antigen, [AK018009]	2.5
Crem	cAMP responsive element modulator. [AK016156]	7.3
Emp1	epithelial membrane protein 1 (Emp1), [NM_010128]	2.3
Fbxl17	Mus musculus 10 days neonate cerebellum cDNA, RIKEN full-length enriched library, clone:B930094M09 product: unknown EST. [AK081162]	5.1
Gas2l3	Mus musculus 16 days embryo head cDNA, RIKEN full-length enriched library, clone:C130036O09 product: unclassifiable, [AK048137]	12.5
Myef2	myelin basic protein expression factor 2, repressor (Myef2), [NM_010852]	10.6
Nrip1	nuclear receptor interacting protein 1 (Nrip1), [NM_173440]	6.2
Pla2g12a	phospholipase A2, group XIIA (Pla2g12a), [NM_023196]	3.2
Psmd11	Mus musculus adult male testis cDNA, RIKEN full-length enriched library, clone: 1700089D09 product: unclassifiable. [AK007029]	12.3
Rps20	Mus musculus bone marrow macrophage cDNA, RIKEN full-length enriched library, clone:I830013G23 product: hypothetical protein. [AK150667]	5.5
Senp7	SUMO1/sentrin specific protease 7 (Senp7), transcript variant 3, [NM_001003972]	12.3
Slc25a16	solute carrier family 25 (mitochondrial carrier, Graves disease autoantigen), member 16 (Slc25a16), [NM_175194]	2.8
Spire1	spire homolog 1 (Drosophila) (Spire1), transcript variant 2, [NM_176832]	9.5
Tdrd3	Mus musculus adult male olfactory brain cDNA, RIKEN full-length enriched library, clone:6430599B16 product: hypothetical Tudor domain containing protein. [AK078326]	2.9
Tmpo	thymopoietin (Tmpo), [NM_011605]	4.3
Trps1	Mus musculus adult male adrenal gland cDNA, RIKEN full-length enriched library, clone: 7330401C17 product: expressed sequence AI115454, [AK078617]	11.4
Ube2n	ubiquitin-conjugating enzyme E2N (Ube2n), [NM_080560]	2.4
Wtap	Mus musculus 12 days embryo embryonic body between diaphragm region and neck cDNA, RIKEN full-length enriched library, clone: 9430038B09 product: unclassifiable. [AK020459]	14. 2

Fold change: ITD-Flt3^+^ KLS cells derived from CreER-Survivin^fx/fx^ compared to control Survivin^fx/fx^ in two experiments.

**Table:** (c) Genes down regulated in LSC and in ITD-Flt3^+^KSL cells deleted with Survivin (24 genes)

Symbol	Name	Fold change
Arg2	arginase type II (Arg2), [NM_009705]	−2.2
Atp2c1	CALCIUM-TRANSPORTING ATPASE TYPE 2C, MEMBER 1 (EC 3.6.3.8) (ATPASE 2C1) (ATP-DEPENDENT CA2+ PUMP PMR1) homolog	−2.2
Chrac1	chromatin accessibility complex 1,. [AK031914]	−4.3
Dhx30	DEAH (Asp-Glu-Ala-His) box polypeptide 30 (Dhx30), [NM_133347]	−3.1
Dsg2	desmoglein 2, (cDNA clone IMAGE:4036406), [BC034056]	−4.1
Eef1g	eukaryotic translation elongation factor 1 gamma (Eef1g), [NM_026007]	−2.2
Herc5	hypothetical regulator of chromosome condensation (RCC1) containing protei⋯	−2.3
Hmga2	high mobility group AT-hook 2 (Hmga2), [NM_178057]	−5.0
Hmgb1	high mobility group box 1, [BC064790]	−3.4
Hmmr	hyaluronan mediated motility receptor (RHAMM) (Hmmr), [NM_013552]	−3.2
Il15	interleukin 15 (Il15), [NM_008357]	−6.1
Jmy	junction-mediating and regulatory protein (Jmy), [NM_021310]	−4.1
Lrrn1	Mus musculus 10, 11 days embryo whole body cDNA, RIKEN full-length enriched library, clone:2810047E21 product: unclassifiable, full insert sequence. [AK012914]	−4.0
Maff	v-maf musculoaponeurotic fibrosarcoma oncogene family, protein F (avian) (Maff), [NM_010755]	−8.4
Mapk14	mitogen activated protein kinase 14 (Mapk14), [NM_011951]	−2.4
Ncoa7	nuclear receptor coactivator 7, [BC076623]	−8.9
Nedd1	neural precursor cell expressed, developmentally downregulated gene 1 (Nedd1), [NM_008682]	−2.6
Parp11	poly (ADP-ribose) polymerase family, member 11 (Parp11), [NM_181402]	−2.5
Pawr	PRKC, apoptosis, WT1, regulator (Pawr), [NM_054056]	−2.8
Rps4x	ribosomal protein S4, X-linked (Rps4x), [NM_009094]	−2.2
Smarce1	SWI [AK042961]	−2.9
Tpbg	trophoblast glycoprotein, [AK050794]	−4.5
Vwf	Von Willebrand factor homolog (Vwf), [NM_011708]	−5.8
Zbtb20	zinc finger and BTB domain containing 20 (Zbtb20), [NM_019778]	−3.1

Fold change: ITD-Flt3**^+^**   KLS cells derived from CreER-Survivin^fx/fx^ compared to control Survivin^fx/fx^ in two experiments.

**Table:** (d) Genes upregulated in LSC and in ITD-Flt3^+^KSL cells deleted with Survivin (37 genes).

symbol	Name	fold change
Cenpa	Mus musculus 0 day neonate eyeball cDNA, RIKEN full-length enriched library, clone:E130306P06 product: hypothetical protein, [AK165029]	10.7
Clcn3	chloride channel 3 (Clcn3), transcript variant c, [NM_173876]	5.5
Cpd	carboxypeptidase D (Cpd), [NM_007754]	6.2
Cul4b	cullin 4B,. [AK164640]	4.9
Ddx52	DEAD (Asp-Glu-Ala-Asp) box polypeptide 52 (Ddx52), [NM_030096]	3.3
Dnajc1	DnaJ (Hsp40) homolog, subfamily C, member 11 (Dnajc11), [NM_172704]	6.4
Ephb2	Eph receptor B2 (Ephb2), [NM_010142]	3.0
Gnaq	guanine nucleotide binding protein, alpha q polypeptide (Gnaq), [NM_008139]	17.2
Gripap1	premature mRNA for mKIAA1167 protein [AK173119]	2.2
Il1rap	interleukin 1 receptor accessory protein (Il1rap), transcript variant 2, [NM_134103]	11.0
Itsn2	SH3 domain protein 1B, [AK161743]	4.4
Lass6	longevity assurance homolog 6 (S. cerevisiae) (Lass6), [NM_172856]	2.3
Lpp	caseinolytic protease, ATP-dependent, proteolytic subunit homolog (E. coli) (Clpp), [NM_017393]	2.1
Mppe1	metallophosphoesterase 1 (Mppe1), [NM_172630]	3.4
Mrps5	mitochondrial ribosomal protein S5, [AK047438]	9.0
Myh9	myosin heavy chain IX (Myh9), [NM_181327]	2.8
Nck2	non-catalytic region of tyrosine kinase adaptor protein 2 (Nck2), [NM_010879]	4.8
Nin	microtubule associated serine/threonine kinase-like (Mastl), [NM_025979]	7.6
Nmt2	N-myristoyltransferase 2, [AK049483]	2.3
Orc5l	origin recognition complex, subunit 5 homolog (S. cerevisiae), [AK054452]	4.6
Osbpl3	Mus musculus adult male aorta and vein cDNA, RIKEN full-length enriched library, clone:A530055M08 product: unknown EST, [AK040984]	8.7
Paqr3	Mus musculus adult male pituitary gland cDNA, RIKEN full-length enriched library, clone:5330440B03 product: unknown EST, [AK030634]	4.4
Pcbd2	pterin 4 alpha carbinolamine dehydratase/dimerization cofactor of hepatocyte nuclear factor 1 alpha (TCF1) 2 (Pcbd2), [NM_028281]	4.2
Rab18	RAB18, member RAS oncogene family (Rab18), [NM_181070]	6.3
Rab8b	RAB8B, member RAS oncogene family (Rab8b), [NM_173413]	2.7
Rae1	RAE1 RNA export 1 homolog (S. pombe), (cDNA clone MGC:73449 IMAGE:5718934), complete cds. [BC060072]	3.3
Sfrs6	splicing factor, arginine/serine-rich 6 (Sfrs6), [NM_026499]	2.2
Sla	src-like adaptor (Sla), [NM_009192]	2.7
Slc31a1	solute carrier family 31, member 1 (Slc31a1), [NM_175090]	4.2
Snx11	sorting nexin 11, [AK037747]	2.6
Sos1	Son of sevenless homolog 1 (Drosophila) (Sos1), [NM_009231]	2.4
Ssh2	mKIAA1725 protein [AK173243]	4.6
Stk3	serine/threonine kinase 3 (Ste20, yeast homolog) (Stk3), [NM_019635]	9.7
Taf1b	TATA box binding protein (Tbp)-associated factor, RNA polymerase I, B (Taf1b), [NM_020614]	4.3
Vps13d	PREDICTED: Mus musculus vacuolar protein sorting 13D (yeast), transcript variant 2 (Vps13d), [XM_001002241]	8.4
Wdr26	PREDICTED: Mus musculus WD repeat domain 26, transcript variant 1 (Wdr26), [XM_977731]	3.3
Xpo4	exportin 4 (Xpo4), [NM_020506]	6.9

Fold change: ITD-Flt3**^+^** KLS cells derived from CreER-Survivin^fx/fx^ compared to control Survivin^fx/fx^in two experiments.

**Table 2:** Genes regulated by Survivin in ITD-Flt3^+^KSL cells that are preferentially expressed in HSC.

symbol	dysregulation in LSC
2310057J16Rik	
2610002J02Rik	
2610042L04Rik	
4930533K18Rik	
9130011E15Rik	
Adrb3	
Arl3	
Atp1b1	
AW112010	
Ccnd1	
Cdk9	
Chst1	
Crem	dysregulated in LSC
Cyp2e1	
Dnmt3b	
Emp1	dysregulated in LSC
Erbb2ip	
Fmn2	
Fnbp4	
Foxb1	
Gabpb1	
Glycam1	
Gpr56	
Hmga2	dysregulated in LSC
Ifi203	
Kcnj6	
Kcnmb1	
Lama5	
Lrrn1	dysregulated in LSC
Ltbr	
Maff	dysregulated in LSC
Mup3	
Myef2	dysregulated in LSC
PCP4	
Prox1	
Rps4x	dysregulated in LSC
Scd2	
Slc22a9	
Slit2	
Sos1	dysregulated in LSC
Stx3	
Tcf3	
Traf1	
Wnt6	
Xrcc1	

## References

[1] Altieri DC (2003). Survivin, versatile modulation of cell division and apoptosis in cancer. *Oncogene*.

[2] Altieri DC (2003). Validating survivin as a cancer therapeutic target. *Nature Reviews Cancer*.

[3] Fukuda S, Pelus LM (2006). Survivin, a cancer target with an emerging role in normal adult tissues. *Molecular Cancer Therapeutics*.

[4] Fukuda S, Foster RG, Porter SB, Pelus LM (2002). The antiapoptosis protein survivin is associated with cell cycle entry of normal cord blood CD34^+^ cells and modulates cell cycle and proliferation of mouse hematopoietic progenitor cells. *Blood*.

[5] Fukuda S, Pelus LM (2002). Elevation of Survivin levels by hematopoietic growth factors occurs in quiescent CD34+ hematopoietic stem and progenitor cells before cell cycle entry. *Cell Cycle*.

[6] Fukuda S, Pelus LM (2001). Regulation of the inhibitor-of-apoptosis family member survivin in normal cord blood and bone marrow CD34^+^ cells by hematopoietic growth factors: implication of survivin expression in normal hematopoiesis. *Blood*.

[7] Fukuda S, Mantel CR, Pelus LM (2004). Survivin regulates hematopoietic progenitor cell proliferation through p21WAF1/Cip1-dependent and—independent pathways. *Blood*.

[8] Leung CG, Xu Y, Mularski B, Liu H, Gurbuxani S, Crispino JD (2007). Requirements for survivin in terminal differentiation of erythroid cells and maintenance of hematopoietic stem and progenitor cells. *Journal of Experimental Medicine*.

[9] Carter BZ, Milella M, Altieri DC, Andreeff M (2001). Cytokine-regulated expression of survivin in myeloid leukemia. *Blood*.

[10] Adida C, Recher C, Raffoux E (2000). Expression and prognostic significance of survivin in de novo acute myeloid leukaemia. *British Journal of Haematology*.

[11] Badran A, Yoshida A, Wano Y (2003). Expression of the anti-apoptotic gene survivin in myelodysplastic syndrome. *International Journal of Oncology*.

[12] Gilliland DG, Griffin JD (2002). The roles of FLT3 in hematopoiesis and leukemia. *Blood*.

[13] Levis M, Murphy KM, Pham R (2005). Internal tandem duplications of the FLT3 gene are present in leukemia stem cells. *Blood*.

[14] Fukuda S, Singh P, Moh A (2009). Survivin mediates aberrant hematopoietic progenitor cell proliferation and acute leukemia in mice induced by internal tandem duplication of Flt3. *Blood*.

[15] Velculescu VE, Madden SL, Zhang L (1999). Analysis of human transcriptomes. *Nature Genetics*.

[16] Majeti R, Becker MW, Tian Q (2009). Dysregulated gene expression networks in human acute myelogenous leukemia stem cells. *Proceedings of the National Academy of Sciences of the United States of America*.

[17] Kelly LM, Liu Q, Kutok JL, Williams IR, Boulton CL, Gilliland DG (2002). FLT3 internal tandem duplication mutations associated with human acute myeloid leukemias induce myeloproliferative disease in a murine bone marrow transplant model. *Blood*.

[18] Huang DW, Sherman BT, Lempicki RA (2009). Systematic and integrative analysis of large gene lists using DAVID bioinformatics resources. *Nature Protocols*.

[19] Cline MS, Smoot M, Cerami E (2007). Integration of biological networks and gene expression data using Cytoscape. *Nature Protocols*.

[20] Ivanova NB, Dimos JT, Schaniel C, Hackney JA, Moore KA, Lemischka IR (2002). A stem cell molecutar signature. *Science*.

[21] Asanuma K, Tsuji N, Endoh T, Yagihashi A, Watanabe N (2004). Survivin enhances fas ligand expression via up-regulation of specificity protein 1-mediated gene transcription in colon cancer cells. *The Journal of Immunology*.

[22] Takizawa BT, Uchio EM, Cohen JJ, Wheeler MA, Weiss RM (2007). Downregulation of survivin is associated with reductions in TNF receptors’ mRNA and protein and alterations in nuclear factor kappa B signaling in urothelial cancer cells. *Cancer Investigation*.

[23] Balkhi MY, Christopeit M, Chen Y, Geletu M, Behre G (2008). AML1/ETO-induced survivin expression inhibits transcriptional regulation of myeloid differentiation. *Experimental hematology*.

[24] Salz W, Eisenberg D, Plescia J (2005). A survivin gene signature predicts aggressive tumor behavior. *Cancer Research*.

[25] Kostrouchova M, Kostrouch Z, Saudek V, Piatigorsky J, Rall JE (2003). BIR-1, a Caenorhabditis elegans homologue of Survivin, regulates transcription and development. *Proceedings of the National Academy of Sciences of the United States of America*.

[26] Chen J, Jin S, Tahir SK (2003). Survivin enhances aurora-B kinase activity and localizes aurora-B in human cells. *The Journal of Biological Chemistry*.

[27] Bolton MA, Lan W, Powers SE, McCleland ML, Kuang J, Stukenberg PT (2002). Aurora B kinase exists in a complex with survivin and INCENP and its kinase activity is stimulated by survivin binding and phosphorylation. *Molecular Biology of the Cell*.

[28] Nowak SJ, Corces VG (2000). Phosphorylation of histone H3 correlates with transcriptionally active loci. *Genes and Development*.

[29] Li J, Lin Q, Yoon H-G (2002). Involvement of histone methylation and phosphorylation in regulation of transcription by thyroid hormone receptor. *Molecular and Cellular Biology*.

[30] Cheung P, Tanner KG, Cheung WL, Sassone-Corsi P, Denu JM, Allis CD (2000). Synergistic coupling of histone H3 phosphorylation and acetylation in response to epidermal growth factor stimulation. *Molecular Cell*.

[31] Wei Y, Yu L, Bowen J, Gorovsky MA, David Allis C (1999). Phosphorylation of histone H3 is required for proper chromosome condensation and segregation. *Cell*.

[32] Alvarez RH, Valero V, Hortobagyi GN (2010). Emerging targeted therapies for breast cancer. *Journal of Clinical Oncology*.

[33] Harris TJR, McCormick F (2010). The molecular pathology of cancer. *Nature Reviews Clinical Oncology*.

[34] Hahn CK, Berchuck JE, Ross KN (2009). Proteomic and genetic approaches identify Syk as an AML target. *Cancer Cell*.

[35] Boehrer S, Adès L, Braun T (2008). Erlotinib exhibits antineoplastic off-target effects in AML and MDS: a preclinical study. *Blood*.

